# The Rationale for Growth Hormone Therapy in Children with Short Stature

**DOI:** 10.4274/jcrpe.2017.S003

**Published:** 2017-12-30

**Authors:** Annalisa Deodati, Stefano Cianfarani

**Affiliations:** 1 University of Rome Tor Vergata, Bambino Gesù Children’s Hospital, Dipartimento di Pediatria Universitario Ospedaliero, Rome, Italy; 2 Karolinska Institutet, Department of Women’s and Children’s Health, Stockholm, Sweden

**Keywords:** Growth hormone treatment, idiopathic short stature, small for gestational age

## Abstract

Growth hormone (GH) was first isolated from cadaver pituitary glands, requiring laborious and expensive collection of glands, followed by extraction and purification of the hormone. This limited supply restricted its use to children with severe GH deficiency who were treated with low dosages and suboptimal schedules. The development of recombinant DNA-derived GH, allowed the production of virtually unlimited amounts of GH, leading to the approval for therapy for a large number of childhood conditions characterized by non-GH deficient short stature. The aim of this review is to provide a critical overview on the daily use of GH in two paradigmatic conditions of non-GH deficient short stature which are children born small for gestational age and with idiopathic short stature, highlighting the available strength of evidence for efficacy and safety.

## INTRODUCTION

Short stature is the most common cause of referral to pediatric endocrinology units, though the vast majority of short children have variants of growth such as constitutional delay of growth and puberty (CDGP) and familial short stature (FSS) (1,2,3,4).

Due to the shortage of human growth hormone (GH) prepared by extraction from pituitaries obtained at autopsy, for almost three decades GH therapy was limited to children with the diagnosis of GH deficiency (GHD) (5). Since 1985, when biosynthetic GH was first produced on a large-scale (6,7,8,9,10,11,12,13), the virtually unlimited availability led to a rapid expansion of clinical trials to study the effect of GH in various conditions associated with short stature but with normal GH secretion (14,15). One of the first conditions characterized by non-GH deficient short stature which was nevertheless treated with GH was Turner syndrome (TS) (16). The preliminary short-term trials, though reporting encouraging results, raised doubts about appropriateness and long-term effectiveness and safety (17). Other genetic syndromes such as Noonan syndrome (18) and achondroplasia (19,20) were considered as potential indications for GH therapy. Most of these pioneering studies with biosynthetic GH in non-GH deficient short children were short-term trials that considered the increase in height velocity after 6-12 months of GH therapy as the main outcome measure for assessing GH efficacy.

Following the publication of results from long-term trials, showing efficacy and safety of GH therapy, indications for such therapy have been expanded in the last two decades. Although the most frequent condition treated with GH still remains GHD, other growth-related indications for GH treatment are TS, short stature homeobox-containing (SHOX) gene deficiency, Noonan syndrome, Prader-Willi syndrome, growth failure associated with chronic renal insufficiency, short stature in children born small for gestational age (SGA) who do not demonstrate catch-up growth and idiopathic short stature (ISS) (Table 1).

Therefore, the initial GH replacement therapy limited to GH deficient patients has metamorphosed into a pharmacological therapy to include different conditions of non-GH deficient short stature. The rationale of this treatment is based on the empiric observation of growth acceleration in response to GH administration, rather than on a pathophysiological approach. From a biological perspective, the close relation between GH dose and response to therapy, in terms of growth acceleration, is well established and confirms the clinical finding of excessive height gain in children with hypersecretion of GH-the more GH, the more growth.

The aim of this review is to provide a critical overview on the daily use of GH in two paradigmatic conditions of non-GH deficient short stature, namely SGA and ISS.

### Small for Gestational Age

Children born SGA are at risk of becoming short adults. Although most children born SGA show catch-up growth in the first 24 months of life, approximately 10% remain below the 3^rd^ centile throughout childhood and adolescence and into adulthood (21). To date, however, the mechanisms underlying postnatal catch-up growth in children born SGA are still largely unknown (22). Birth length is a more important predictor of adult height than birth weight (22,23,24,25) and though genetics play a key role in controlling the growth trajectory, the endocrine mechanisms underlying early growth remain undetermined.

SGA refers to the size at birth and is defined as a birth weight and/or length of at least two standard deviation (SD) scores (SDS) below the mean for gestational age and gender (26,27). The etiology of intrauterine growth retardation ultimately leading to SGA consists of a broad spectrum of maternal, environmental, placental and fetal factors, but in a significant proportion of cases the reason for being born SGA remains unclear.

SGA newborns show high circulating levels of GH and low concentrations of both insulin-like growth factor 1 (IGF-1) and IGF-binding-protein-3 which normalize in the first months of postnatal life, thus suggesting a transient GH insensitivity (28,29). In childhood and adolescence, SGA subjects show normal GH responses to stimulation tests (30). Alterations in diurnal GH secretion profile have been reported by isolated studies but are of limited diagnostic and prognostic utility (31,32). On average, both IGF-1 and IGF-binding protein-3 levels are reduced in SGA children by approximately one SD, but the individual variability is wide, indicating broad heterogeneity in the underlying endocrine and non-endocrine mechanisms.

Genetic abnormalities in the GH-IGF axis such as IGF-1 and IGF-1 receptor gene deletions and point mutations have been associated with small size at birth and severe postnatal growth retardation (33,34,35).

The first short term trials with pituitary derived GH in short SGA children date back more than 50 years (36,37). More recently, a promising short-term trial with biosynthetic GH (38) paved the way for long-term studies whose results led to the approval from regulatory authorities such as the Food and Drug Administration (FDA) in 2001 and European Medicines Agency (EMA) in 2003, although with slightly different criteria. FDA approval includes a dose of 0.48 mg/kg per week for treatment of children born SGA who fail to manifest catch-up growth by the age of two years, whereas EMA approved GH for the treatment of short children born SGA after the age of four years at a dose of 0.22 mg/kg per week.

A consensus conference organized by the main international societies of Pediatric Endocrinology and the Growth Hormone Research Society proposed that children born SGA with height less than minus 2.5 SDS at the age of two years or with height less than minus 2 SDS at the age of four years should be eligible for GH treatment. The dose should range from 35 to 70 mg/kg per day, with the higher dose to be preferred for those with more severe growth retardation (30).

**Figure 1:**
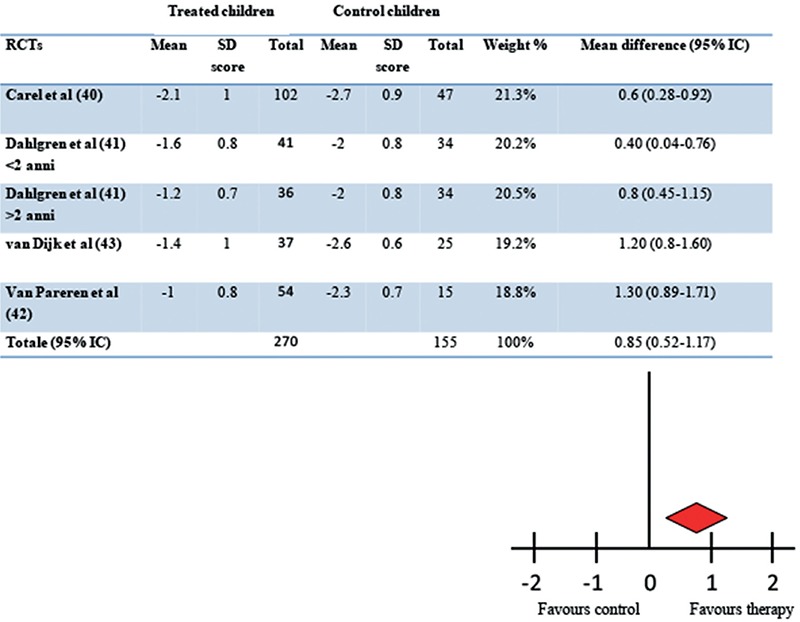
Effect of long term growth hormone therapy on adult height in randomised controlled trials. Results of meta-analysis according to random model (39) in children born small for gestational age. The mean difference in adult height between treated and untreated children was 0.85 standard deviation (IC 95% 0.52-1.17, p<0.001) SD: standard deviation, RCTs: randomized controlled trials

The improvement of adult height is unanimously considered the best outcome measure of the efficacy of GH therapy in SGA. The approval of this indication was based on the results from a few randomized controlled trials (RCTs) conducted until the achievement of adult height. Moreover, the available data were collected from small study cohorts treated with different treatment regimens. We set out to critically evaluate the strength of evidence by performing a systematic review and meta-analysis of all the available trials (39). The results of this meta-analysis showed that from an initial number of 29 studies reporting the effect of GH therapy in SGA children, only four RCTs were conducted up to the achievement of adult height, and these four studies included a total of 391 children (40,41,42,43). The mean adult height of the GH-treated group exceeded controls by 0.85 SDS (5.7 cm) after eight years of therapy. Furthermore, no significant difference in efficacy was observed between the two GH dose regimens (33 vs. 67 mg/kg per day) (Figure 1). A wide individual variability in response to GH therapy was present in all studies, consistent with the heterogeneity of conditions underlying SGA. The quality grading of the studies was performed according to Endocrine Society criteria (44) and revealed that all four RCTs had moderate quality evidence.

Although there is a large body of evidence suggesting that low birth weight is associated with a high risk of developing insulin resistance, glucose intolerance and metabolic disorders in later life, thus far GH treatment in SGA children has not been associated with major side effects. A transient insulin resistance, increased fasting glucose and reduced tolerance during oral glucose-tolerance testing have been reported (42,43,45,46). Longer follow-up of SGA subjects treated with GH during childhood, up to six years after discontinuation of therapy, showed a similar body composition, insulin sensitivity, blood pressure and a more beneficial lipid profile compared with untreated, short, young adults born SGA (47,48,49).

GH treatment has been reported not to influence the age at onset and progression of puberty, regardless of the dose (50) and duration of puberty and pubertal height gain are apparently not affected by the use of higher doses of GH (50,51,52). Moreover, GH therapy seems to improve body composition and cardiovascular profiles in children born SGA, reducing fat mass, blood pressure and lipid levels and increasing lean body mass (46,53). Even intelligence, psychosocial functioning and quality of life (QOL) have been reported to improve during GH therapy in SGA children (54,55,56).

In general, GH therapy is not indicated in SGA during adolescence due to the reduced growth potential remaining after entering puberty. However, combined therapy with GH and gonadotropin releasing hormone analogs (administered for two years) has recently been reported to be safe and effective in improving adult height in SGA children with more severe growth retardation at the onset of puberty (57,58).

Children with Silver-Russell syndrome (SRS) constitute a syndromic subgroup of SGA and were classically considered to be less, or even non-responsive, to GH therapy (30). Smeets et al (59) have recently reported that SRS children are significantly shorter than non-SRS SGA children at start of GH therapy but gain more height during treatment, resulting in a similar height SDS at onset of puberty in SRS and non-SRS. Thereafter, there is a decline in height SDS from puberty onset to adult height attainment in SRS compared to non-SRS, leading to a significantly shorter adult height (59). However, although SRS children do not attain the same adult height as non-SRS, the total height gain is similar suggesting a positive growth promoting effect of such therapy (59). In addition, a positive effect of GH therapy on body composition, motor development, appetite and reduced risk of hypoglycemia has been reported (59,60).

Wide individual variability in response to GH therapy has been reported in all studies. Multiple linear regression analyses were used to construct the best model for predicting adult height SDS. The major predictors of adult height reported so far are: (i) height and weight at the start of GH treatment; (ii) target height; (iii) pretreatment growth rate; and (iv) prepubertal years treated with GH (61,62).

Is GH therapy a panacea for children born SGA? Although the results of most studies strongly encourage GH treatment in SGA children, it has to be pointed out that (i) the longitudinal follow-up is still relatively short; (ii) the overall number of children enrolled in the trials is relatively small, but, more importantly, (iii) almost all studies have been performed by the same group of investigators in the Netherlands, thus leaving open the question about the replicability of their results in other geographical and scientific contexts.

### Idiopathic Short Stature

The story of GH treatment in children with ISS begins in 1983 with the first trial conducted with pituitary derived GH in 15 non-GH deficient short children who were treated for six months (15). In all children, growth rate increased by more than 2.0 cm per year during treatment. This short-term study paved the way for a series of trials which led to FDA approval for such an indication in 2003.

ISS is defined as a condition in which the height of an individual is more than 2 SDS below the corresponding mean height for a given age, sex and population, without evidence of systemic, endocrine, nutritional, or chromosomal abnormalities (63,64). Therefore, children defined as having ISS have a normal size at birth and normal GH secretion. ISS is defined by criteria rather than a diagnosis per se and encompasses a variety of conditions including both mild skeletal abnormalities not falling into any of the known, classified disorders and non-syndromic genetic conditions, as well as normal variants of growth such as CDGP and FSS (65).

In 2003, GH therapy was approved in the United States for children with ISS with height at or less than -2.25 SDS (1.2 percentile) below the mean for age and sex, associated with growth rates unlikely to permit attainment of adult height in the normal range, and in whom diagnostic work up excluded other causes for short stature that should be observed or treated by other means. A consensus conference of the International Societies of Pediatric Endocrinology and the Growth Hormone Research Society proposed that children with ISS whose heights are less than -2.0 SDS and who are more than 2.0 SDS below their mid-parental target height or had a predicted height less than -2.0 SDS warrant consideration for treatment (63).

However, controversy still exists about the degree of effectiveness of GH therapy in children with ISS (66). A preliminary systematic review of literature showed that one year of GH therapy induced an acceleration of growth velocity and suggested that long-term GH therapy was able to increase adult height (67). However, this systematic review did not consider the outcome measures analytically and did not evaluate and classify the trials according to the quality of evidence and strength of recommendation. Furthermore, at that time, the review could take into account the results of only one randomized control trial, limited to eight girls followed up to the achievement of adult height (68). The authors, cautiously and wisely concluded that the focus of assessment should increasingly shift from efficacy in promoting growth to effectiveness in promoting health and well-being as a function of increased growth (67).

**Figure 2:**
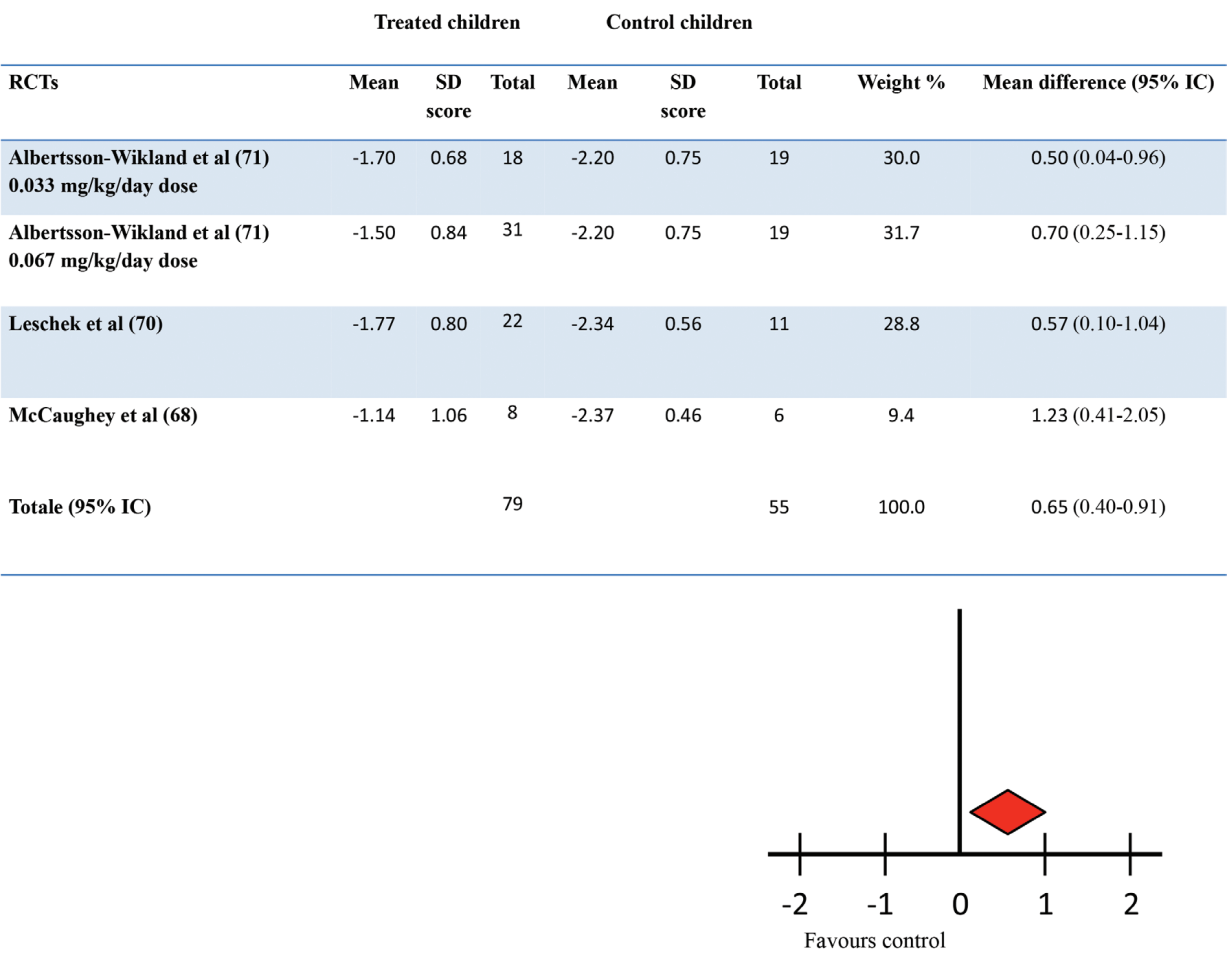
Effect of long term growth hormone therapy on adult height in randomised controlled trials. Results of meta-analysis according to random model (69) in children with idiopathic short stature (ISS). The mean difference in adult height between treated and untreated ISS children was 0.65 standard deviation (IC 95% 0.4-0.91, p<0.001) SD: standard deviation, RCT: randomized controlled trial

A more detailed and updated meta-analysis of available trials, including quality grading according to the Endocrine Society criteria which classifies the quality of evidence into one of four categories (high, moderate, low and very low) (44) was performed by the authors of this review in 2011 (69). The aim was to systematically determine the impact of GH therapy on adult height of children with ISS. This systematic review of the literature showed that from an initial number of 19 long-term trials, only ten met the criteria of controlled trials. Three RCTs (including 115 children, 79 cases and 36 controls) (68,70,71) and seven non-RCTs (including 477 children, 181 cases and 296 controls) (72,73,74,75,76,77,78) reported data on adult height. Two randomized clinical trials were classed as of moderate quality evidence and one of low quality evidence. Six non-randomized clinical trials were classed as of low quality evidence and one of low-moderate quality evidence. The adult height of the GH treated children exceeded, on average, that of the controls by 0.65 SD (about 4 cm) (Figure 2). In the seven non-RCTs, the adult height of the GH treated group exceeded, on average, that of the controls by 0.45 SDS (about 3 cm).

The main conclusions were that: (i) no single, high quality evidence, RCT was carried out up to the achievement of adult height; (ii) that the overall magnitude of GH effect in reducing the adult height deficit in children with ISS was on average less than that achieved in other conditions for which GH was licensed and; (iii) that the individual response to therapy was highly variable (69).

More recently, van Gool et al (79) have reported that high dose GH therapy in prepubertal children with ISS does not improve adult height, as it increases height gain during treatment but, at the same time, accelerates bone maturation, resulting in a similar adult height compared with the untreated controls.

Finally, the effect on adult height of a combined therapy with GH plus gonadotropin-releasing hormone analogs in ISS adolescents with relatively early puberty was assessed. The modest results in height gain led the authors to advise physicians against the use of this treatment in clinical practice (80).

Because estrogens mediate skeletal maturation and epiphyseal fusion, aromatase inhibitors have been used to delay bone maturation. The first trial with aromatase inhibitors was performed in boys with CDPG with apparently promising results (81,82) and, afterwards in children with ISS alone (83) or in combination with GH (84,85). These still preliminary results indicate that aromatase inhibitors, especially in combination with GH, seem to be effective in stimulating growth. However, caution is needed as potential adverse effects include reduced high-density lipoprotein cholesterol, increased insulin resistance, vertebral deformities, impairment of cognitive function and long-term effects on spermatogenesis and infertility (86). Therefore, the use of aromatase inhibitors must be considered experimental and to be performed only in strictly controlled clinical trials.

Wide individual variability in the response to GH therapy was reported in all clinical trials conducted in ISS children. The major predictors of adult height reported so far were: (i) early age at start of therapy; (ii) dose of GH; (iii) length at birth; (iv) difference between height and mid-parental height and; (v) delay in bone age (87).

In conclusion, the available evidence suggests that long-term GH therapy reduces the adult height deficit in children with ISS. The still open question is whether this treatment is worthwhile considering the impact of the height gained on physical and psychosocial wellbeing, burden for patients and parents, potential adverse effects, cost of therapy and patients’/parents’ expectations.

### Final Remarks

The available evidence shows that GH therapy can increase adult height in non-GH deficient short children born SGA or with ISS. However, in both conditions the efficacy is far less than in GHD. A critical review of available data shows that to date, no study has fulfilled the evidence based medicine criteria for high quality of evidence and strong recommendation. The individual response to therapy is highly variable and further studies are needed to identify what defines the responders.

The assumption underlying the pharmacological use of GH in non-GH deficient short children is that GH treatment, by increasing adult height, improves the QOL of subjects with short stature. However, data are conflicting and inconclusive, and this potential effect cannot be considered at the moment as a strong argument for such therapy (55,88,89,90,91,92,93,94,95,96).

The long-term safety of GH therapy has recently been questioned by observational studies reporting increased risk of mortality and morbidity in young adults treated with GH during childhood (97,98). Although these data have not been confirmed (99,100,101), continued surveillance of subjects exposed to recombinant human GH is essential both during treatment and in the years after treatment cessation (102,103).

Finally, further high-quality evidence from randomised, double blind, placebo controlled trials up to the achievement of adult height would be necessary to determine the real efficacy, ideal dosage and long term safety of GH therapy in non-GH deficient short children.

## Figures and Tables

**Table 1:**
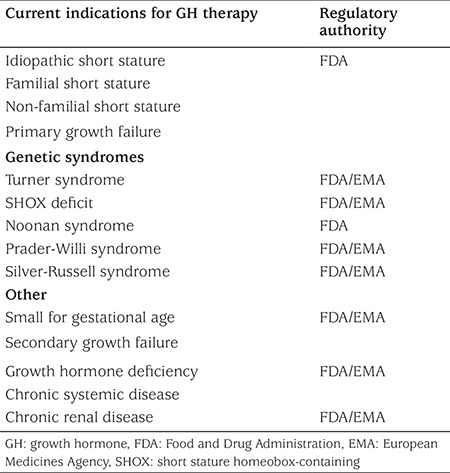
Indications approved by Food and Drug Administration and European Medicines Agency for growth hormone therapy
